# The Combination of Tissue-Engineered Blood Vessel Constructs and Parallel Flow Chamber Provides a Potential Alternative to In Vivo Drug Testing Models

**DOI:** 10.3390/pharmaceutics13030340

**Published:** 2021-03-05

**Authors:** Wanjiku Njoroge, Andrea C. Hernández Hernández, Faiza Idris Musa, Robert Butler, Alan G. S. Harper, Ying Yang

**Affiliations:** 1 School of Pharmacy and Bioengineering, Keele University, Stoke-on-Trent ST4 7QB, UK; w.njoroge@keele.ac.uk (W.N.); andreac.2h@gmail.com (A.C.H.H.); f.i.musa11@gmail.com (F.I.M.); 2 Department of Cardiology, Royal Stoke Hospital, Stoke-on-Trent ST4 6QG, UK; rob.butler@uhnm.nhs.uk; 3 School of Medicine, Keele University, Staffs ST5 5BG, UK

**Keywords:** cardiovascular disease, ketamine, atorvastatin, platelets, endothelial progenitor cells, test tissue models

## Abstract

Cardiovascular disease is a major cause of death globally. This has led to significant efforts to develop new anti-thrombotic therapies or re-purpose existing drugs to treat cardiovascular diseases. Due to difficulties of obtaining healthy human blood vessel tissues to recreate in vivo conditions, pre-clinical testing of these drugs currently requires significant use of animal experimentation, however, the successful translation of drugs from animal tests to use in humans is poor. Developing humanised drug test models that better replicate the human vasculature will help to develop anti-thrombotic therapies more rapidly. Tissue-engineered human blood vessel (TEBV) models were fabricated with biomimetic matrix and cellular components. The pro- and anti-aggregatory properties of both intact and FeCl_3_-injured TEBVs were assessed under physiological flow conditions using a modified parallel-plate flow chamber. These were perfused with fluorescently labelled human platelets and endothelial progenitor cells (EPCs), and their responses were monitored in real-time using fluorescent imaging. An endothelium-free TEBV exhibited the capacity to trigger platelet activation and aggregation in a shear stress-dependent manner, similar to the responses observed *in vivo*. Ketamine is commonly used as an anaesthetic in current in vivo models, but this drug significantly inhibited platelet aggregation on the injured TEBV. Atorvastatin was also shown to enhance EPC attachment on the injured TEBV. The TEBV, when perfused with human blood or blood components under physiological conditions, provides a powerful alternative to current in vivo drug testing models to assess their effects on thrombus formation and EPC recruitment.

## 1. Introduction

Cardiovascular diseases are among the leading causes of mortality and morbidity worldwide. These are caused by aberrant platelet activation caused by endothelial dysfunction and exposure of plasma to collagen- and tissue factor-rich atherosclerotic plaques. However, it is not possible to study this practically or ethically in human patients. Most studies assessing the effect of drugs on cardiovascular disease rely on animal models to predict and explain their effects in humans [1]. Different animal species have been used to evaluate certain features of cardiovascular disease, such as zebrafish, pigs, rabbits, and rodents. Mice have become the animal of choice for disease modelling given their genetic similarity to humans, their fast breeding rate, and well-established methods for creating genetic knock-outs [2,3]. Additionally, intravital microscopy allows the real-time examination of thrombus formation on artificial vessel injuries in response to ferric chloride (FeCl_3_) or laser injury [4]. These arterial thrombosis models have become popular for examining the molecular mechanisms underlying thrombus formation and how these can be impacted by drug treatments.

The interpretation of the data obtained from murine thrombosis models is complicated by the use of anaesthetics. A survey of investigators performing intravital microscopy in murine thrombosis models found that ketamine, xylazine, and pentobarbital are the most commonly used anaesthetics [5]. However, previous studies have demonstrated that each of these anaesthetics can have an inhibitory effect on platelet function [5,6,7]. For instance, ketamine inhibited platelet aggregation through the suppression of IP_3_ formation and also by inhibiting thromboxane synthase activity [7,8]. Additionally, ketamine can also interfere with endothelial nitric oxide production, as well as smooth muscle Ca^2+^ signalling [9,10]. This suggests that the use of ketamine in intravital microscopy studies could create a baseline inhibition of platelet function as well as modulation of normal haemostatic properties of the vessel wall, which could overestimate the effect of genetic knockouts or drug treatments on normal haemostatic responses.

These shortcomings provide an opportunity to create alternative thrombosis models by recreating normal haemostatic conditions by flowing human blood through human tissue-engineered arterial constructs. Tissue-engineered arteries were initially produced to use as alternatives to autologous vessels for vascular grafting. Vascular tissue engineering was pioneered in 1986 by Weinberg and Bell, who generated the first tissue-engineered blood vessels (TEBVs) by culturing vascular cells on a collagen-based scaffold [11]. Nearly 40 years later, there are few TEBVs currently being used in clinical application. However, great progress has been made in improving the biomimicry of TEBVs. A number of previous studies have demonstrated that it is possible to generate tissue-engineered arteries through a variety of methods that can withstand normal arterial blood flow conditions whilst replicating the functional properties of the native arteries [12,13,14]. This has been achieved through using a variety of approaches including the use of a variety of scaffold material both synthetic (e.g., polyvinyl alcohol and gelatin [15]) and natural extracellular matrix molecules (collagen, elastin [16]). The properties of these scaffolds can be further modified to increase their mechanical strength through compression or chemical crosslinking or made more porous by freeze-drying [16]. Furthermore, the use of bioreactors has been influential in producing ideal culture conditions for vascular cells to ensure they assume the cellular phenotypes found in vivo [17]. These approaches have created TEBVs that possess a number of desirable properties such as the ability to support physiological spiral laminar flow [15], to mechanically withstand physiological arterial blood pressures [15,16], and to support the growth of a healthy endothelial cell lining [17]. This is consistent with the key parameters required for a substrate for clinical vascular grafting. These studies have commonly assessed the ability of the TEBVs to withstand activation of haemostatic and inflammatory responses of blood cells flowing through them. However, to utilise TEBVs as an animal-free alternative to current in vivo arterial thrombosis models requires a demonstration that they are capable of eliciting appropriate cellular reactions upon damage and that drug treatments are able to modulate that response.

Previously, we have utilised tissue engineering approaches to create human arterial models that replicate the normal haemostatic properties of the intimal and medial lining of human arteries [18]. This includes the use of an electrospun polylactic acid (PLA) nanofiber scaffold to create an intimal layer construct that provides contact guidance to ensure that the endothelial cells can be aligned in the direction of flow, similar to the native artery. The medial layer construct is formed by human coronary artery smooth muscle cells cultured within a collagen hydrogel. Musa et al. (2016) showed that their tissue-engineered blood vessels are able to replicate the anti- and pro-aggregatory properties of native arteries when the intimal layer is intact and absent, respectively [18]. Our real-time spectrofluorimetry measurements of cytosolic Ca^2+^ signalling provided a sensitive method to assess platelet activation upon exposure to the tissue-engineered constructs. These results clearly demonstrate that the intimal, medial, and full blood vessel constructs replicate the in vivo ability to modulate platelet function [18]. The thrombus formation upon the surface of the construct indicated by aggregated DiOC_6_-labelled platelets under a fluorescent microscope can be visualized in the consequent examination. However, these studies were performed under non-physiological mixing conditions. We have not previously examined the ability of the constructs to support thrombus formation under physiologically relevant shear stress from perfusion of platelets. The flexibility of a layer-by-layer fabrication approach in tissue engineering in conjunction with a perfusion device offers a great opportunity to study endothelial dysfunction and repair mechanisms. Endothelial function is an important and independent predictor for the severity of cardiovascular disease. An impaired endothelium is a key driver in the development of cardiovascular disease [19]. Circulating bone marrow-derived endothelial progenitor cells (EPCs) have been found to correlate to endothelial function and to aid in neovascularisation and re-endothelialisation of injured vessels, maintaining vascular function and homoeostasis [19,20]. In models of myocardial infarctions and arterial injury, EPCs have been shown to localize preferentially to sites of vascular lesions, after which they divide, proliferate, and become incorporated into the endothelial layer of existing vessels, and promote the outgrowth of new vascular networks. These cells also have an effect on surrounding cells by producing angiogenic growth factors [21,22].

The most common drugs used to treat/prevent the development of cardiovascular disease are statins, with atorvastatin being the most well-known. This drug has been in use for decades, and its effects have been extensively studied. Some of these include reduction of the accumulation of esterified cholesterol into macrophages, increase of endothelial nitric oxide (NO) synthase, reduction of the inflammatory process, increased stability of the atherosclerotic plaques, and restoration of platelets activity and of the coagulation process [23,24]. Despite the known pleiotropic actions of atorvastatin, there is currently limited data on the impact this drug has on EPC ability to mediate endothelium repair.

In this study, we aimed to examine whether our tissue-engineered (TE) human arterial models were able to mimic the pro- and anti-aggregatory properties of the damaged and intact artery under physiological flow conditions. We also aimed to examine whether our tissue-engineered arterial constructs could support EPC recruitment and whether this could be modulated by drug treatments. This was achieved by incorporating the constructs within a commercially available parallel-plate flow chamber and perfusing them with washed human platelet suspensions at arterial shear stresses. We examined whether this biomimetic test model system could be used as a potential alternative to in vivo drug testing models in thrombosis and EPC homing. This system was used to perfuse platelets and various cell populations over the TE constructs at physiologically relevant or pathological shear stress, allowing the real-time profiling of their interactions, and for the evaluation of changes in both the surface and structure of the blood vessel, as well as changes in the perfusate. The impact of ketamine on platelet activation and the effect of atorvastatin on EPC homing when EPC being exposed to TEBVs with a FeCl_3_-induced lesion were investigated. Through these studies, we demonstrated that human tissue-engineered arterial constructs, when perfused with human blood or freshly prepared washed human platelet suspension under physiological conditions, provide a human model system that can be used to study the effect of drugs without the potential confounding impact of species differences and use of anaesthetics.

## 2. Materials and Methods

### 2.1. Fabrication of 3D Tissue-Engineered Blood Vessel Constructs

Fabrication of 3D tissue-engineered intimal layers (TEILs), media layers (TEMLs), and the complete tissue-engineered blood vessel was achieved using human umbilical vein endothelial cells (HUVECs) and human cardiac artery smooth muscle cells (HCASMCs), both obtained from GIBCO, Life Technologies. Cells were cultured with medium 200 and 231, respectively, also obtained from GIBCO, Life Technologies, and used between passage numbers 2 and 5. The construction of the TEIL, TEML, and TEBV constructs was performed using our previously described methodology [18], as such these protocols are outlined briefly below.

### 2.2. Electrospinning

Aligned nanofibers were made by dissolving Poly-l,d-lactic acid (96% L/4% D, inherent viscosity of 5.21 dL/g, Purac BV, Gorinchem, the Netherlands) (PLA) in a 7:3 mixture of chloroform and dimethylformamide (DMF) (Sigma, Welwyn Garden City, UK) into 2% solution. The operational parameters of nanofiber fabrication followed the established protocol [25]. In brief, this 2% PLA solution was deposited onto detachable metal collectors, comprised of two partially insulated steel blades (30 cm × 10 cm), and connected to a permanent copper plate with a steel wire. The two steel blades had a gap of 5 cm between them where the fibers were deposited. Deposition of the fibers involved connecting the permanent plate to a negative electrode, and a syringe containing the solution was connected to a positive electrode. The PLA was extruded through an 18G needle and delivered at a rate of 0.025 mL/min. The electrodes were electrified with a power supply charged at ±6 kV (Spellman HV, Pulborough, UK). Nanofibers were collected and affixed onto acetate frames and were sterilized by UV irradiation thrice per side before use in culture. The nanofiber diameter was measured as ~500 µm and the mat thickness ~3 µm [25]. The porosity of the mat was smaller than 1 µm since no endothelial cells were observed to migrate through the nanofiber layer [18].

### 2.3. TEML Assembly

To create TEML constructs, HCASMCs, at a density of 5 × 10^5^ cells /mL, were mixed with neutralized 3 mg/mL type I collagen (Corning) solution. Two hundred microlitres of this solution was loaded onto 0.5 cm × 2.0 cm filter paper frames, which fit the dimensions of the parallel-plate flow chamber. The formed TEML constructs were used when the HCASMCs attained typical spindle-shaped morphology and reached confluence. TEMLs were cultured in whole medium 231, with media changes every 2 days for up to 10 days.

### 2.4. TEIL Assembly

To prepare TEIL constructs, a neutralized acellular collagen gel (3 mg/mL), having the same dimensions detailed above, was formed first. Once the gel set, aligned PLA nanofibers [18], coated in 10 ng/mL fibronectin, were placed on the surface of the gel. HUVECs were then seeded at a density of 2 × 10^5^ cells/mL on the nanofibers. The TEIL samples were cultured in whole medium 200, with media changes every 2 days, for 10 days to allow attainment of normal cell morphology and surface area coverage.

### 2.5. TEBV Assembly

**Figure 1:**
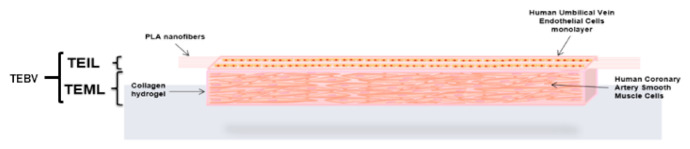
Schematic representation of a 3D human tissue-engineered blood vessel (TEBV). The diagram shows the structure of the tissue-engineered (TE) construct and the locations of the cells within the intimal (TEIL) and medial layer (TEML). The medial layer adopts an eventual thickness of approximately 3 mm while the intimal layer atop the nanofibers is approximately 30 μm.

This model was a combination of the TEIL and TEML. TEML was created first and HUVECs seeded, as previously described, on fibronectin-coated PLA nanofibers after HCASMCs attained desired spindle-shaped morphology. The complete TEBV was returned to culture with HCASMC and HUVEC whole media mixed 7:3. The schematic for TEML, TEIL, and TEBV assembly is shown in Figure 1.

### 2.6. Perfusion System

#### 2.6.1. Perfusion Chamber

**Figure 2:**
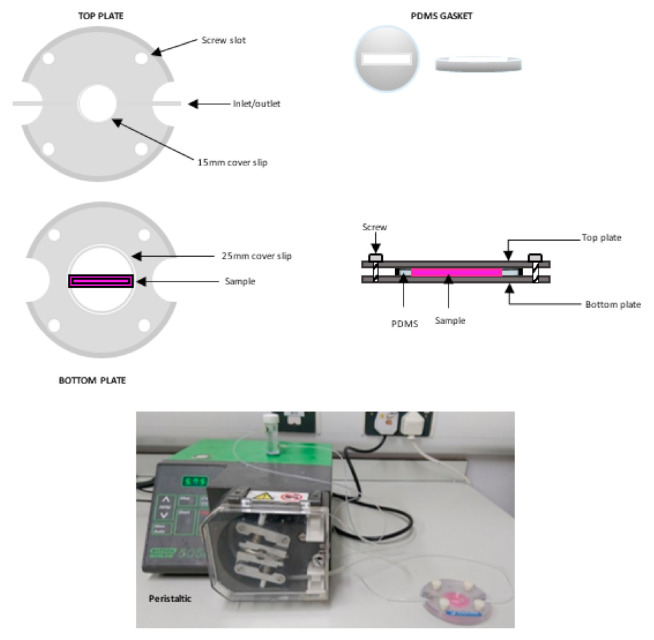
Schematic drawing and image illustrating the perfusion chamber assembly. The sample is aligned along the flow path between the inlet and outlet on the bottom plate. The outlet, affixed with silicone grease to the top plate, is aligned in the same way and placed over the sample. The assembled chamber is connected to a reservoir and a peristaltic flow pump.

To generate peristaltic flow and a range of shear stress values, a peristaltic pump (Watson-Marlow 505 series, Watson-Marlow Fluid Technology, Falmouth, UK) was used along with a commercially sourced parallel-plate flow chamber (ProFlow chamber; Warner Instruments, Hamden, CT, USA) modified as outlined below. Media to be perfused was contained in a 7 mL reservoir connected to the pump and flow chamber using polyethylene (PE-90) tubing as shown in Figure 2.

#### 2.6.2. Perfusion Gaskets

To facilitate the perfusion of our 3D vascular models, a specialized gasket was created. The gasket was manufactured using polydimethylsiloxane (PDMS). A circular ring with a diameter of 30 mm was first cut, then a 25 mm (length) × 5 mm (width) × 3 mm (depth) rectangular opening in the centre of the circle (Figure 2).

#### 2.6.3. Parallel-Plate Flow Chamber and Shear Stress

The assembled flow chamber was used to generate laminar flow to exert physiological shear stress on the intimal surface of the TEBV. The dimensions of the gasket used comprise and determine the dimensions of the flow chamber. The equation used to determine the shear stress generated on the endothelial surface of the TE constructs is:

$$\tau =\frac{6uQ}{bh^{2}}$$

where *u* is the viscosity of the fluid being perfused (1.5 Cp), *Q* being the flow rate (*Q*; either 0.077 cm^3^/s and 0.007 cm^3^/s), *b* the width of the gasket opening (5 mm), and *h* being the height between the upper surface of the construct and top plate of the chamber (2.5 mm). The two fluid-flow rates used provided shear stresses of 22.2 dyne/cm^2^ and 2.2 dyne/cm^2^ for the performed experiments which are consistent with arterial and venous shear rates respectively [26,27].

### 2.7. Lesion Models

To mimic vascular injury, a FeCl_3_ lesion was created on the TEBV by dipping a 1 mm^2^ square of filter paper in 10% FeCl_3_ and placing this onto the upper surface of the TE constructs for 1 min. After this, the TE constructs were washed with PBS/HBS to eliminate excess FeCl_3_, then topped up with fresh media. This mode of lesioning was also applied to TE constructs for EPC perfusion.

### 2.8. Platelet Preparation

This study was approved by the Keele University (UK) Research Ethics Committee (MH-200155, 1 May 2018). Blood was donated by healthy, drug-free volunteers who gave written informed consent. Blood was obtained by venepuncture. The blood was mixed with acid citrate dextrose (ACD; 85 mM sodium citrate, 78 mM citric acid, and 111 mM D-glucose) at a ratio of 5:1. Platelet-rich plasma (PRP) was obtained by soft descent centrifugation at 725 g for 8 min. After centrifugation, the PRP was collected and treated with aspirin (50 mM) and apyrase (0.1 U/mL). PRP was again centrifuged at 450 g for 20 min, then resuspended in supplemented HEPES-buffered saline (HBS; pH 7.4, 145 mM NaOH, 10 mM HEPES, 10 mM D-glucose, 5 mM KCl, 1 mM MgSO_4_) to reach a platelet density of 2 × 10^8^ cells/mL. The HBS was supplemented with 1 mg/mL bovine serum albumin (BSA), 1.8 mg/mL glucose, 0.1 U/mL apyrase, and 200 µM CaCl_2_. Prior to perfusion, the assembled perfusion system without the TE construct was perfused with 1% BSA solution for 1 h to prevent unwanted platelet adhesion to the components of the perfusion system. The gasket and the spacers were incubated overnight with 1% BSA solution at room temperature.

### 2.9. EPC Isolation and Culture

EPCs were isolated by collecting 60 mL of whole blood from healthy volunteers. To prevent coagulation, the blood was split into 2 falcon tubes with 5 mL of ACD in each. The blood-anticoagulant mix was then split into 15 mL falcon tubes and centrifuged at 700 g for 8 min. After centrifugation, the sequence of layers occurred as follows (seen from top to bottom): plasma, enriched cell fraction (interphase consisting of lymphocytes/peripheral bone marrow cells (PBMCs), erythrocytes, and granulocytes. The plasma fraction was carefully discarded, leaving approximately 0.5–1 mL above the interface. The enriched cell fraction was pooled into one tube and diluted 1:1 with PBS. To further separate the cell, i.e., eliminate residual plasma and Red Blood Cells (RBCs), the pooled fraction was carefully layered over 8 mL of Ficoll-Paque, ensuring no mixing of the layers, and centrifuged again for 20 min at 400 g. After centrifugation, any residual red blood cells are below the separation medium, and the enriched cell fraction should be immediately above it with diluted plasma and platelets above this. After isolation, the cell-rich fraction was diluted with PBS, then centrifuged again at 400 g for 10 min. After centrifugation, the supernatant was discarded, and the resultant pellet was resuspended in 2 mL of complete EPC media. The cell suspension was then split into 2 wells of a 12-well plate that had been coated with 2.5 μg/cm^2^ fibronectin. On day 1, the contents of the wells were agitated and transferred to new wells. This was repeated for the next 3 days. Media was changed daily for the first 7 days then every 2–3 days for up to 20 days.

### 2.10. Cell Labelling

#### 2.10.1. CFSE

Carboxyfluorescein succinimidyl ester (CFSE) dye was used to label EPC cells at a concentration of 2 µL/mL of cell suspension. Cells were incubated for 15 min at 37 °C, then centrifuged for 3 min at 300 g. The pellet was re-suspended in 5 mL of fresh supplemented media. The cell suspension was allowed to rest for 30 min at 37 °C before loading into the perfusion system.

#### 2.10.2. DiOC_6_

To facilitate visualization of platelet adhesion and aggregation upon the TE constructs under flow conditions, platelets were labelled with DiOC_6_, a fluorescent membrane dye. Blood was mixed 5:1 with ACD (anticoagulant). The anticoagulant was mixed with the membrane dye to make a final concentration of 1μM, prior to the addition of whole blood. This mixture was then incubated for 10 min at room temperature, then centrifuged to obtain PRP. Centrifugation was done at 1500 g for 8 min. The resultant PRP was then treated with 100 μM aspirin and 0.1 U/mL apyrase. This was followed by a centrifugation wash at 350 g for 20 min. The platelet pellet was then re-suspended with supplemented HBS, creating a final cell density of 2 × 10^8^ cells/mL.

### 2.11. Ketamine Treatment

To assess if the platelet responses might be affected by ketamine treatment, experiments were performed in which both platelets and the TE constructs were pre-treated with ketamine before the perfusion of platelets on TE constructs. TEMLs were incubated with 1 mM ketamine (Narketan) or HBS for 1 h at 37 °C, after which they were perfused with washed DiOC_6_-labelled platelets incubated with either 300 µM ketamine or an equivalent volume of the vehicle, or HBS, under the same conditions stated above. Platelet aggregation on the perfused TE constructs was evaluated by fluorescence microscopy (Leica MSV269) under excitation wavelength of 485 nm and emission of 501 nm. As a static comparative, TE constructs were treated with 1 mM ketamine and placed in a cuvette with DiOC_6_-labelled platelets treated with ketamine or HBS for 15 min at 37 °C. Whilst untreated TEML constructs were placed atop untreated platelet samples as the control.

### 2.12. Platelet Aggregometry

Platelet aggregometry was performed using a modification of the previously published technique [28]. Following platelet incubation with TE constructs, 200 µL of the platelet suspension was transferred into a 96-well plate and then placed into a plate reader prewarmed to 37 °C (BioTek Synergy 2 microplate, Winooski, VT, USA). Baseline absorbance readings were taken once at a wavelength of 600 nm, obtaining an absolute absorbance reading post TE construct incubation. In the present assay, the plate reader was set up to use a fast-shaking mode between absorbance readings to aid in sample mixing.

### 2.13. Atorvastatin Treatment

Following FeCl_3_ lesioning, the TE constructs were incubated with 60 μg/mL atorvastatin calcium trihydrate (Sanofi) for 3 and 5 h at 37 °C. This was followed by the perfusion of CFSE-labelled EPCs (without atorvastatin in EPC perfusate as control) for 45 min in the parallel-plate chamber. Images were taken using a Leica inverted microscope (Leica MSV269) under an excitation wavelength of 485 nm and emission of 501 nm. Cell attachment was quantified with ImageJ.

### 2.14. Statistics and Data Analysis

Values stated are mean ± SEM of the number of observations (n) indicated. Analysis of statistical significance was performed using a two-tailed Student’s *t*-test as well as two-way analysis of variance (ANOVA), confirmed using the Brown–Forsythe test. *p* < 0.05 was considered statistically significant.

## 3. Results

The adhesion and aggregation of platelets by exposure to the TE constructs under dynamic flow conditions were assessed by monitoring fluorescence from DiOC_6_-labelled human platelets upon the surface of the TE constructs (solid phase activation), as well as monitoring activation of platelets remaining within the solution by platelet aggregometry (liquid phase activation). Three types of TE constructs were evaluated, TEIL, TEML, and TEBV, with the acellular collagen hydrogel as a negative control as we have previously demonstrated this to not elicit platelet activation [18]. The homing effect of drug atorvastatin on EPC attachment on these TE constructs under dynamic flow conditions was assessed.

### 3.1. Cells in TE Constructs Attained Typical Morphology and Organisation

**Figure 3:**
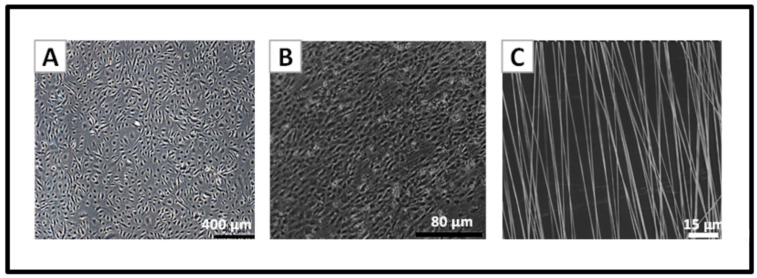
Cellular morphology for TEIL constructs. (**A**) Human umbilical vein endothelial cells (HUVECs) cultured for 4 days in a culture flask. (**B**) HUVECs cultured on the nanofibers for 10 days, (**C**) SEM picture of aligned PLA nanofibers.

Consistent with our previous findings, it was possible to generate TEIL, TEML, and TEBV constructs with HUVECs and HCASMCs showing typical normal cellular morphology when grown atop (HUVECs) or within (HCASMCs) the collagen hydrogel scaffold using our previously published layer-by-layer fabrication technique [18]. Figure 3 demonstrates the effect of aligned nanofibers on HUVEC alignment, with cells showing more organised growth/orientation compared to culture flasks. Meanwhile, smooth muscle cells maintain spindle-shaped morphology while embedded in the collagen gel (data not shown [18]).

### 3.2. TEML Supports Shear-Dependent Platelet Aggregation under Physiological Flow Conditions

**Figure 4:**
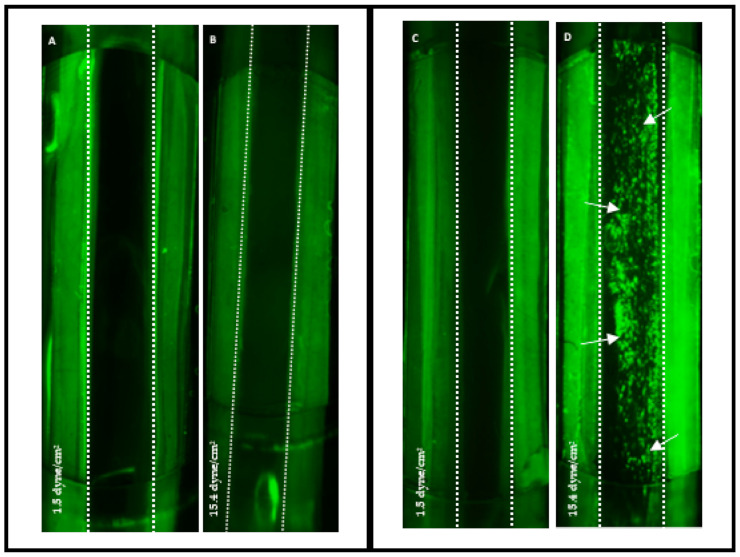
Fluorescence images of tissue-engineered media layer (TEML) constructs after exposure to platelets perfusion at different shear stress rates. DiOC_6_-labelled human platelets suspension was perfused through a parallel-plate flow chamber over the surface of acellular collagen gels (**A**,**B**) and TEML (**C**,**D**) for 15 min at venous (**A**,**C**) (2.2 dyne/cm^2^) and arterial (**B**,**D**) (22.2 dyne/cm^2^) shear stresses. White dotted line denotes the central hydrogel area. Area on either side of the dotted line is the filter paper frame. Arrows highlight aggregated platelets.

We first assessed whether the TEML is able to support platelet aggregation under two different shear stresses indicative of those found in the arterial (22.2 dyne/cm^2^) and in the venous circulation (2.2 dynes/cm^2^; [29]). The TEML construct is a model for an endothelium-denuded blood vessel and therefore can be used to assess pro-aggregatory properties of the medial layer. Figure 4A,B show that acellular collagen gels did not trigger platelets’ activation under both low and high shear stress rate, consistent with our previous findings under static conditions [18]. When the TEML constructs were exposed to platelets perfused at venous shear stresses, sporadic platelet adhesion was observed (Figure 4C). The strong platelet aggregation observed under arterial shear stresses demonstrates that shear stress and fluid flow influence platelet adhesion (Figure 4D). These studies are consistent with a number of previous studies that have demonstrated a role for shear-dependent platelet aggregation in driving thrombus formation [30,31].

The platelets adhered and significantly aggregated when exposing to the media layer of the constructs (Figure 4D). Since no aggregation was observed on the acellular hydrogels (Figure 4A,B), platelet aggregation should be triggered by neo-collagen produced by the embedded HCASMCs. This corresponded well with previous studies that demonstrated that the pro-aggregatory properties of the TEML were mainly attributed to the native collagen secretion of the SMCs [32] and is consistent with our previous work under stirred conditions [18]. Additional synthesis and secretion of thrombogenic molecules may also contribute to platelet activation and aggregation.

### 3.3. TEBVs Prevent Platelet Aggregation under Physiological Flow Conditions

The endothelial lining of the native human artery produces platelet inhibitors such as nitric oxide (NO) and prostaglandins to prevent thrombosis. Upon vascular damage, the loss of the antithrombotic endothelial lining, and the exposure of the prothrombotic properties of the medial layer, triggers thrombus formation. To test whether the TEBV is able to replicate this endothelium-dependent modulation of the haemostatic properties of the construct under physiological flow conditions, TEBVs with differences in the integrity of the endothelial cell layer were exposed to human platelet suspension under arterial shear stresses. In these experiments, we used (i) a TEBV with a fully confluent endothelial layer, (ii) a TEBV with a partially confluent endothelial layer, and (iii) a TEBV in which an intimal injury was triggered with FeCl_3_, a common injury model used in murine thrombosis models [33].

**Figure 5:**
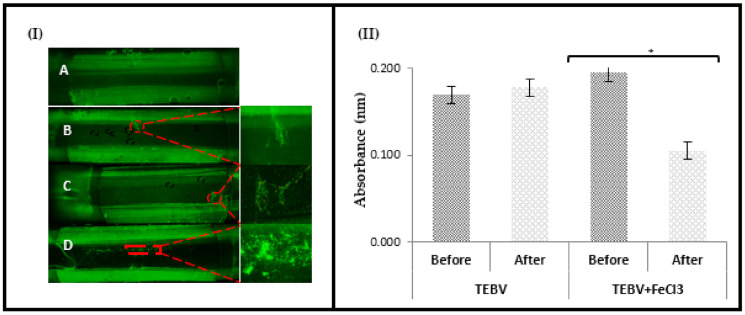
Characterisation of tissue-engineered blood vessel (TEBV) constructs with full, partial, and impaired intimal layer after exposure to platelets perfusion for 15 min at a shear stress of 22.2 dynes/cm^2^. (**I**) The fluorescence images of DiOC_6_-labelled human platelet suspension which was perfused through a parallel flow chamber over the surface of the TEBV. (**A**) TEBV construct cultured for 10 days with a confluent endothelial cell layer. (**B**,**C**) TEBV with a partial endothelial layer coverage, 4-day culture, and (**D**) with impairment of the endothelial layer by FeCl_3_ incubation. The green clumps over the construct surface indicate platelet aggregation and single platelet adhesion. Higher magnification images of the platelet aggregates are shown in the inserts. (**II**) The aggregometer analysis of the aggregation state of the platelet solution before and after exposure to tissue-engineered blood vessels (TEBVs). The data represent mean ± SEM; *n* = 3. Significance is indicated by (*), *p* ≤ 0.01.

Real-time fluorescence imaging revealed that TEBVs with an intact, confluent endothelial layer did not show platelet aggregation upon their surfaces over a 15 min period of perfusion (Figure 5I (A)). In contrast, the TEBV with a partially confluent endothelial layer exhibited limited platelet aggregation. Platelets adhered only in areas that were not covered with endothelial cells, allowing their direct contact with the media layer. However, not all the areas lacking endothelial coverage showed platelet aggregation, suggesting that the endothelial cells on the TEIL may be capable of secreting sufficient anti-thrombotic molecules to prevent platelet aggregation (Figure 5I (B,C)). Dramatic contrast observations of platelets’ aggregation behaviours on FeCl_3_-lesioned TEBV samples were revealed as shown in Figure 5I (D), in which massive platelet aggregates are attached to the constructs. The insert picture shows the dense morphology of the aggregated platelets, and the low magnification image shows the overall localisation of the aggregates. The strip-like aggregate location was larger than the lesion area (1 mm^2^), implying that cytokines produced by lesioned endothelial layer and exposed subendothelial proteins (collagens in our model) could be circulated away from the lesion site, triggering aggregation away from the lesion site. FeCl_3_-mediated endothelial cell injury allowed exposure of platelets to the pro-aggregatory medial layer in the construct, providing a reliable FeCl_3_-triggered arterial injury model.

These qualitative results correspond well with the comparison of the quantitative aggregation state of the platelets before and after perfusion, in which FeCl_3_ injury could be seen to trigger aggregation of platelets within the platelet suspension (Figure 5II). In regards to the integrity of the endothelial layer, it can be observed that measurements of the absorbance, before and after perfusion, do not present a significant difference in the TEBV with a full endothelial layer. This indicates that platelet aggregation is prevented by the presence of an intact endothelial layer. On the contrary, significant differences in platelet aggregation were observed in FeCl_3_-treated TEBVs. This is consistent with platelets producing and releasing autocrine activators to recruit platelets to the growing thrombi, thus triggering platelet aggregation within the platelet suspension.

### 3.4. Ketamine Inhibited Platelet Aggregation at Arterial Shear Stress

**Figure 6:**
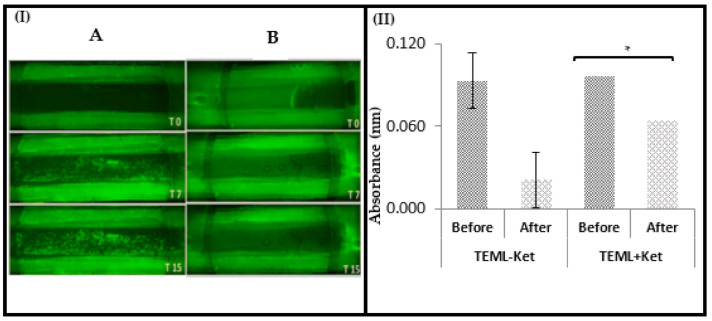
Characterisation of perfused platelet solution to the surface TEML incubated with and without ketamine under physiological shear strain. (**I**) The fluorescence images of DiOC_6_-labelled human platelet suspension which was perfused through a parallel flow chamber over the surface of the TEML. Human platelet suspensions were treated with 300 µM ketamine or an equivalent volume of its vehicle, HBS, and perfused over the TEML for 15 min using a parallel-plate flow chamber at 22.2 dynes/cm^2^. Images were recorded in real-time over the course of 15 min. (**A**) Untreated sample; (**B**) Ketamine-treated sample. (T0) Beginning of perfusion; (T7) 7 min into perfusion; (T15) 15 min into perfusion. (**II**) Aggregometer analysis of the aggregation state of the perfused platelet solution. Aggregation states of platelets untreated (left) and pre-treated (right) with ketamine before and after perfusion over TEML surface. Error bars represent SEM. Significance is indicated by (*), *p* ≤ 0.05.

The data above demonstrate that the TEML, and the FeCl_3_-treated TEBV, trigger platelet aggregation through the exposure of the pro-aggregatory medial layer in the endothelial denuded section of these constructs. Thus, our TEBV perfusion system can be used in conjunction with the FeCl_3_ injury model, which is commonly used in murine thrombosis models [33]. As our human TEBV model does not require the addition of anaesthetics to our blood samples, experiments were performed to assess if the addition of ketamine, the most commonly used anaesthetic in murine thrombosis models, could artificially alter the platelet aggregatory responses seen. As the TEML provides a more pronounced aggregatory response due to the absence of an endothelial lining, we used this system to investigate whether ketamine could impact thrombus formation by human platelets under physiological flow conditions. DiOC_6_-labelled platelets were treated with 300 µM ketamine prior to perfusion over the TEML surface under arterial shear stress (22.2 dynes/cm^2^). Significant platelet aggregation was found in the TEML perfused at high shear stress without ketamine treatment (Figure 6). The platelets treated with ketamine were found to lose their ability to adhere to the TEML surface. These results indicate that ketamine-treated platelets (Figure 6I (B)) were less reactive than untreated platelets (Figure 6I (A)), which showed significant aggregation on the surface. Ketamine not only significantly inhibited platelet aggregation on the construct surface but also inhibited their activation within the surrounding platelet suspension, as demonstrated by the aggregation state of platelets before and after exposure to the TEML (Figure 6II). This is likely due to the known effect of ketamine inhibiting platelet Ca^2+^ signalling [34,35]. This would prevent dense granule secretion, which would in turn prevent the activation of these cells in the perfused platelet suspension.

**Figure 7:**
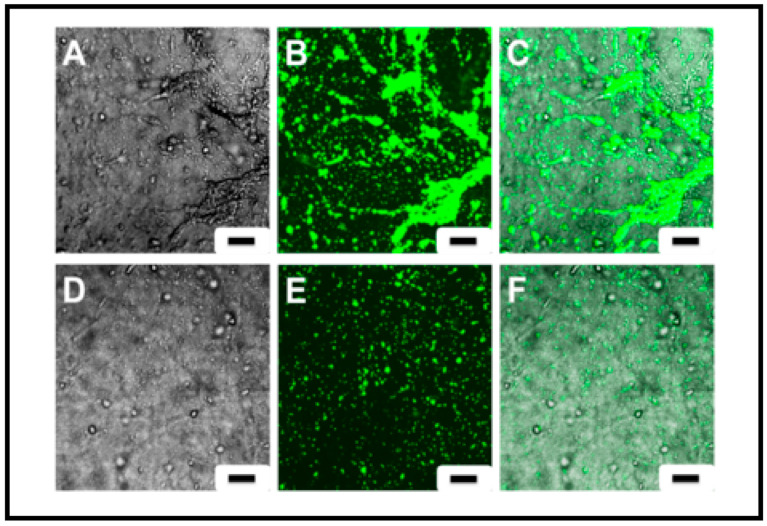
Visible and fluorescence images illustrating the state of platelet aggregation after TEML incubation either with or without ketamine. Constructs were first incubated with 1 mM ketamine, at 37 °C, for 1 h then a 15 min incubation, also at 37 °C, with 1 mL of washed platelets that were pre-treated with 1 mM ketamine. Following the second incubation, platelets were washed, and aggregates on the surface were imaged with a fluorescent microscope. Images (**A**–**C**): untreated samples. Images (**D**–**F**): ketamine-treated samples. Results are representative of 4 experiments, *n* = 6. Scale bar = 100 µm.

To further confirm the inhibitory effect of ketamine on platelets, the platelets treated or untreated with ketamine were exposed to corresponding TEMLs in cuvette holders under continuous magnetic stirring. Representative images are shown in Figure 7.

Fluorescent imaging of the TEML surface exposed to DiOC_6_-labelled platelets showed that samples treated with ketamine had fewer platelet aggregates appearing on the TEML surface (Figure 7D–F). The untreated samples (Figure 7A–C) displayed greater adhesion, as well as formation of multiple platelet aggregates.

### 3.5. Atorvastatin Increases EPC Attachment

**Figure 8:**
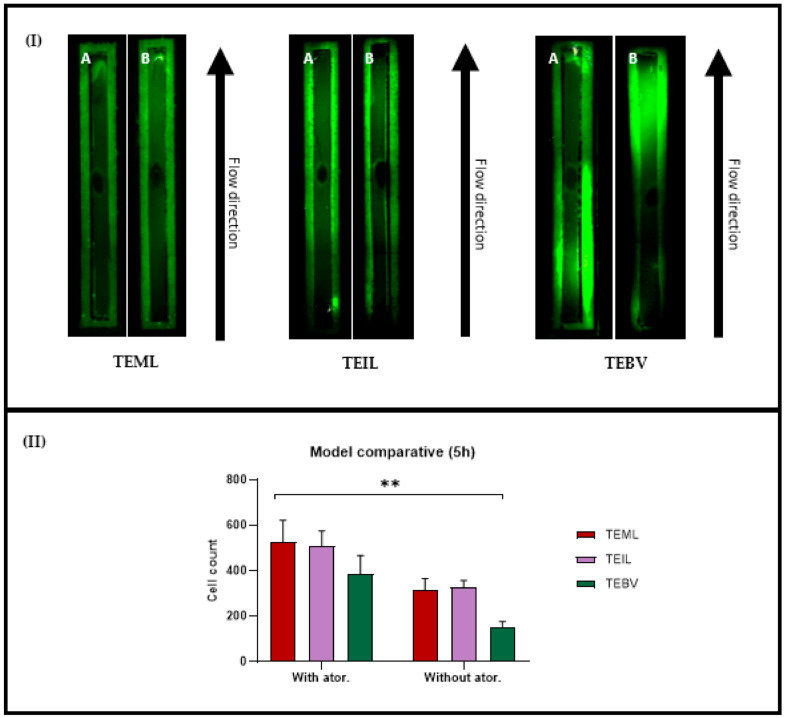
Characterisation of perfusion and attachment of endothelial progenitor cells on TE constructs with and without 60 μg/mL atorvastatin (5 h pre-incubation). (**I**) Representative carboxyfluorescein succinimidyl ester (CFSE)-labelled EPC perfusion images. For all models (TEML, TEIL, TEBV), A is with atorvastatin, B is without atorvastatin. Green dots are EPCs. (**II**) The quantification of EPC attachment. Error bars represent SEM, *n* = 3 for all samples. *p* ≤ 0.05. Significance is indicated by (**).

Atorvastatin has been reported to increase circulating numbers of EPCs [23]. To evaluate whether atorvastatin also has an effect on the recruitment and attachment of perfused EPCs, our three TE construct variants were lesioned with FeCl_3_, then incubated with 60 μg/mL atorvastatin for 5 h. Constructs were then perfused with EPCs for 45 min at 22.2 dynes/cm^2^. EPCs were perfused at a density of 1 × 10^4^ cells/mL. After perfusion, constructs were imaged and attached cells quantified (Figure 8).

Across all the models shown here, it was evident that atorvastatin incubation increased the number of cells that attached to the lesioned surfaces of the constructs (Figure 8II). We have previously demonstrated the pro aggregatory properties of the TEML, and Figure 8 suggests that atorvastatin increases these pro-aggregatory properties. The data presented here suggest that without either the endothelial (TEML) or medial (TEIL) layers, the response is stronger than when both layers are present, suggesting a synergistic effect between the medial and intimal layers in terms of modulating cell recruitment. The almost steady state of attachment of EPCs on the TEML suggests that the FeCl_3_ lesion is not the driving factor for cell attachment but rather time-dependent signalling/cytokine production by the representative cells and the presence of atorvastatin.

## 4. Discussion

Being able to rapidly and effectively screen novel and pre-existing drug therapies for the treatment of cardiovascular disease in a human model system would provide a significant advance in our abilities to treat patients at risk of a thrombotic event. In this paper, we demonstrate that our human TEBVs are able to effectively trigger both the activation of thrombus formation and EPC recruitment to vascular injury under physiological flow conditions. Through the use of this humanised experimental system, we should be able to better determine effective drug concentrations and combinations prior to clinical trials. Additionally, it would reduce our need to perform costly, ethically challenging preclinical trials on animals, which require the use of anaesthetics that may significantly impact the results of these trials. Thus, tissue engineered human blood vessel models show promise in improving the translational potential of preclinical studies of drug delivery, drug action, and drug discovery in pharmaceutical research.

Through developing PDMS sample holders and perfusion gaskets, we were able to successfully modify a commercially available parallel flow chamber to incorporate our previously described TEBV. This allowed us to examine whether these constructs are able to support thrombus activation and EPC recruitment under physiological flow conditions. In this study, we confirmed that, similar to static conditions, our acellular type I rat collagen hydrogel is unable to support significant human platelet activation under physiological flow conditions. However, our TEML constructs have been shown to produce type I and III neo-collagen that is able to trigger significant platelet activation [18]. Here we demonstrate that these are able to support significant platelet activation under arterial shear stresses but not under those more typically found in large veins. This is consistent with previous work [36,37] demonstrating that thrombus formation is regulated by shear-dependent platelet aggregation, thus confirming that our TEBV can replicate the normal haemostatic properties of native blood vessels. In contrast, the presence of an intact intimal layer completely blocks the activation of human platelets perfused over the surface of the TEBV. However, when the endothelial layer was impaired, either by incomplete coverage of endothelial cells (via a shortened culture period) or via FeCl_3_-induced injury, the TEBV constructs were able to trigger an effective haemostatic reaction consistent with that seen in the native artery (Figure 5). FeCl_3_-injured TEML also displayed platelet aggregation under flow, an observation that was absent on uninjured constructs. Location of FeCl_3_ application had no impact on this observation, demonstrating that the observed effect is due to endothelial damage caused by FeCl_3_ and not via the presence of FeCl_3_ itself [33,38]. This can be concluded as no enhancement of platelet activation and aggregation observed upon FeCl_3_ treatment of the endothelial-free TEML constructs (data not shown). The data presented here support the traditional explanation that FeCl_3_-induced injury results in thrombus formation in murine arteries through endothelial denudation, facilitating platelet activation upon the exposure of sub-endothelial collagen. These findings demonstrate that this test model can be used to study platelet inhibition and activation in a convenient, operator-friendly, and dynamic manner. The image analysis of the adhered platelets on the TE constructs, and assessment of platelet aggregation in liquid phase using aggregometry, allows the extraction of both qualitative and quantitative datasets to assess ex vivo human thrombus formation.

This study demonstrated that ketamine exerted a strong negative effect on platelet aggregation and activation. This corresponds well with previous in vitro and in vivo studies that have investigated the underlying mechanisms of ketamine’s inhibition of platelet function [6]. In our experiments, we observed that ketamine almost completely inhibits thrombus formation upon the TEML construct. It is possible that this effect is caused by the inhibition of dense granule secretion by ketamine, as autocrine signalling molecules released from here are known to be crucial to the recruitment of circulating platelets onto the surface of forming thrombi [7,39]. This inhibitory effect could artefactually alter the size and structure of thrombi seen in current animal thrombosis models, potentially leading to an overestimation of the effectiveness of putative anti-platelet therapies. This is consistent with previous findings that show that the use of different anaesthetics differentially impacts the efficacy of integrin αIIbβ3 blockers in murine thrombosis models [39], thus providing initial evidence that our model system is a potential alternative to current in vivo studies. A more detailed side-by-side comparison examining the impact of different anaesthetics on the thrombotic response in current in vivo models, and in the TEBV model presented here, will be required to fully validate the model system.

These findings highlight the value of using tissue-engineered human blood vessels for drug testing. By using human cells and eliminating the need for anaesthesia, we should be able to accurately model the processes of haemostasis and vascular repair to improve the translational potential of any findings. Additional advantages of our model include a reduction in the use of animal thrombosis models, elimination of the need for costly intravital microscopy equipment, and a lower cost of TE construct production compared to housing mouse colonies.

We also used the TEBV to demonstrate that atorvastatin enhances recruitment and attachment of perfused EPCs. Although previous studies have demonstrated that atorvastatin increases circulating numbers of EPCs [19], there has been limited study on its ability to modulate EPC recruitment to the damaged vascular wall, which may partly underlie the beneficial effects of this drug in preventing acute cardiovascular events. Possible mechanisms by which atorvastatin might enhance EPC recruitment to the damaged TEBV is via increased bioavailability of NO [40] or activation of matrix metalloproteinase-2 and 9 (MMP2 and MMP9) [41]. It is also possible that the SDF-1 CXCR4 axis is also involved in recruiting EPCs to sites of vascular injury. This theory is supported by the findings by Luo et al., 2018 [42]. They found that NO production was induced by SDF-1, which triggers multiple signalling pathways, resulting in chemokine-induced changes of the EPC cytoskeleton leading to enhanced cell migration [42]. The value of our models is that various doses/concentrations of different drugs, as well as combinations of drugs, can be tested faster than conventional models and also allows for real-time monitoring without measures such as anaesthesia being needed. The ease of assembly also makes it possible to combine multiple cell types and can be adapted for any species. In comparison to other in vitro models [43,44], our models also allow for real-time visualisation of cell attachment due to the nature of the perfusion chamber used, containing both a top and bottom window, as well as the open-faced nature of the models themselves.

## 5. Conclusions

The layer-by-layer assembly of human blood vessel models provides a convenient and reliable research tool to investigate the interaction of blood components, such as platelets and circulated progenitor cells, with a blood vessel. This provides a simple method to assess the impact of a variety of drug interactions on haemostasis and vascular repair. The parallel-plate flow chamber, plus the adjustable dimensions of the PDMS gasket, enabled incorporation of 3D tissues into the chamber, permitting their exposure to perfusion with blood components at different physiological shear stresses. The labelled cells allow the monitoring of cellular activation and adhesion in real-time. The intact, confluent intimal layer can inhibit platelet activation, whilst the partially formed intima did not. The medial layer with newly formed collagen triggered platelet aggregation, whilst collagen gel made from rat skin collagen type I did not. The presence of a similar concentration of ketamine used in current in vivo thrombosis models inhibited the ability of human platelets to adhere to the TEML surface. The tissue-engineered vessels could also be used to demonstrate that atorvastatin is able to enhance the homing capabilities of EPCs by improving their ability to adhere to the damaged tissue-engineered blood vessel under flow conditions. Here we show that the combination of tissue-engineered arterial constructs and a parallel-plate flow chamber can be used to effectively simulate the haemostatic and vascular repair processes ex vivo. Therefore, these results indicate that this model system is able to provide a potential alternative to in vivo testing models. This will also permit us to test the predicted mechanisms of action of a selected anti-thrombotic drug without the need for animal models and may be modified further to simulate other clinical conditions.

## Data Availability

All data in this study have been included in this manuscript.

## References

[1] Benam K.H., Dauth S., Hassell B., Herland A., Jain A., Jang K.-J., Karalis K., Kim H.J., MacQueen L., Mahmoodian R. (2015). Engineered In Vitro Disease Models. Annu. Rev. Pathol. Mech. Dis..

[2] Cesarovic N., Lipiski M., Falk V., Emmert M.Y. (2020). Animals in cardiovascular research. Eur. Hear. J..

[3] Camacho P., Fan H., Liu Z., He J.-Q. (2016). Small mammalian animal models of heart disease. Am. J. Cardiovasc. Dis..

[4] Furie B.C. (2005). Thrombus formation in vivo. J. Clin. Investig..

[5] Janssen B.J.A., De Celle T., Debets J.J.M., Brouns A.E., Callahan M.F., Smith T.L. (2004). Effects of anesthetics on systemic hemodynamics in mice. Am. J. Physiol. Circ. Physiol..

[6] Chang Y., Chen T.-L., Wu G.-J., Hsiao G., Shen M.-Y., Lin K.-H., Chou D.-S., Lin C.-H., Sheu J.-R. (2004). Mechanisms Involved in the Antiplatelet Activity of Ketamine in Human Platelets. J. Biomed. Sci..

[7] Nakagawa T., Hirakata H., Sato M., Nakamura K., Hatano Y., Nakamura T., Fukuda K. (2002). Ketamine suppresses platelet aggregation possibly by suppressed inositol triphosphate formation and subsequent suppression of cytosolic calcium increase. Anesthesiology.

[8] Atkinson P., Taylor D., Chetty N. (1985). Inhibition of platelet aggregation by ketamine hydrochloride. Thromb. Res..

[9] Akata T., Izumi K., Nakashima M. (2001). Mechanisms of direct inhibitory action of ketamine on vascular smooth muscle in mesenteric resistance arteries. Anesthesiology.

[10] Kurebayashi N., Ogawa Y. (2001). Depletion of Ca 2+ in the sarcoplasmic reticulum stimulates Ca 2+ entry into mouse skeletal muscle fibres. J. Physiol..

[11] Weinberg C.B., Bell E. (1986). A blood vessel model constructed from collagen and cultured vascular cells. Science.

[12] Bouten C.C., Dankers P.P., Driessen-Mol A.A., Pedron S., Brizard A., Baaijens F.F. (2011). Substrates for cardiovascular tissue engineering. Adv. Drug Deliv. Rev..

[13] Gui L., Dash B.C., Luo J., Qin L., Zhao L., Yamamoto K., Hashimoto T., Wu H., Dardik A., Tellides G. (2016). Implantable tissue-engineered blood vessels from human induced pluripotent stem cells. Biomaterials.

[14] Rothuizen T.C., Damanik F.F.R., Lavrijsen T., Visser M.J.T., Hamming J.F., Lalai R.A., Duijs J.M.G.J., Van Zonneveld A.J., Hoefer I.E., Van Blitterswijk C.A. (2016). Development and evaluation of in vivo tissue engineered blood vessels in a porcine model. Biomaterials.

[15] Parikh V., Kadiwala J., Bastida A.H., Holt C., Sanami M., Miraftab M., Shakur R., Azzawi M. (2018). Small diameter helical vascular scaffolds support endothelial cell survival. Nanomed. Nanotechnol. Biol. Med..

[16] Buttafoco L., Engbers-Buijtenhuijs P., Poot A.A., Dijkstra P.J., Vermes I., Feijen J. (2006). Physical characterization of vascular grafts cultured in a bioreactor. Biomaterials.

[17] Lee P.-H., Tsai S.-H., Kuo L., Hwang C.-Y., Kuo C.-Y., Yang V.C., Chen J.-K. (2012). A prototype tissue engineered blood vessel using amniotic membrane as scaffold. Acta Biomater..

[18] Musa F.I., Harper A.G., Yang Y. (2016). A Real-Time Monitoring System to Assess the Platelet Aggregatory Capacity of Components of a Tissue-Engineered Blood Vessel Wall. Tissue Eng. Part C Methods.

[19] Oikonomou E., Siasos G., Zaromitidou M., Hatzis G., Mourouzis K., Chrysohoou C., Zisimos K., Mazaris S., Tourikis P., Athanasiou D. (2015). Atorvastatin treatment improves endothelial function through endothelial progenitor cells mobilization in ischemic heart failure patients. Atherosclerosis.

[20] Urbich C., Dimmeler S. (2004). Endothelial progenitor cells: Characterization and role in vascular biology. Circ. Res..

[21] Zhang M., Malik A.B., Rehman J. (2014). Endothelial progenitor cells and vascular repair. Curr. Opin. Hematol..

[22] Kunz G.A., Liang G., Cuculi F., Gregg D., Vata K.C., Shaw L.K., Goldschmidt-Clermont P.J., Dong C., Taylor D.A., Peterson E.D. (2006). Circulating endothelial progenitor cells predict coronary artery disease severity. Am. Heart J..

[23] Sandhu K., Mamas M., Butler R. (2017). Endothelial progenitor cells: Exploring the pleiotropic effects of statins. World J. Cardiol..

[24] Stancu C., Sima A. (2001). Statins: Mechanism of action and effects. J. Cell. Mol. Med..

[25] Yang Y., Wimpenny I., Ahearne M. (2011). Portable nanofiber meshes dictate cell orientation throughout three-dimensional hydrogels. Nanomed. Nanotechnol. Biol. Med..

[26] Malek A.M., Alper S.L., Izumo S. (1999). Hemodynamic shear stress and its role in atherosclerosis. JAMA.

[27] Cunningham K.S., I Gotlieb A. (2004). The role of shear stress in the pathogenesis of atherosclerosis. Lab. Investig..

[28] Chan M.V., Armstrong P.C., Warner T.D. (2018). 96-well plate-based aggregometry. Platelets.

[29] Mongrain R., Rodés-Cabau J. (2006). Role of Shear Stress in Atherosclerosis and Restenosis after Coronary Stent Implantation. Revista Española de Cardiología.

[30] Maxwell M.J., Westein E., Nesbitt W.S., Giuliano S., Dopheide S.M., Jackson S.P. (2006). Identification of a 2-stage platelet aggregation process mediating shear-dependent thrombus formation. Blood.

[31] Rana A., Westein E., Niego B., Hagemeyer C.E. (2019). Shear-Dependent Platelet Aggregation: Mechanisms and Therapeutic Opportunities. Front. Cardiovasc. Med..

[32] Chan-Park M.B., Shen J.Y., Cao Y., Xiong Y., Liu Y., Rayatpisheh S., Kang G.C.-W., Greisler H.P. (2008). Biomimetic control of vascular smooth muscle cell morphology and phenotype for functional tissue-engineered small-diameter blood vessels. J. Biomed. Mater. Res. Part A.

[33] Li W., McIntyre T.M., Silverstein R.L. (2013). Ferric chloride-induced murine carotid arterial injury: A model of redox pathology. Redox Biol..

[34] Lisek M., Zylinska L., Boczek T. (2020). Ketamine and calcium signaling—A crosstalk for neuronal physiology and pathology. Int. J. Mol. Sci..

[35] Wong B.S., Martin C.D. (1993). Ketamine inhibition of cytoplasmic calcium signalling in rat pheochromocytoma (PC-12) cells. Life Sci..

[36] Holme P.A., Ørvim U., Hamers M.J.A.G., Solum N.O., Brosstad F.R., Barstad R.M., Sakariassen K.S. (1997). Shear-induced platelet activation and platelet microparticle formation at blood flow conditions as in arteries with a severe stenosis. Arter. Thromb. Vasc. Biol..

[37] Zhang J.-N., Bergeron A.L., Yu Q., Sun C., McIntire L.V., López J.A., Dong J.-F. (2002). Platelet aggregation and activation under complex patterns of shear stress. Thromb. Haemost..

[38] Li W., Nieman M., Gupta A.S. (2016). Ferric chloride-induced murine thrombosis models. J. Vis. Exp..

[39] Sashindranath M., Sturgeon S.A., French S., Bsc D.D.D.C., Selan C., Freddi S., Johnson C., Cody S.H., Nesbitt W.S., Hamilton J.R. (2019). The mode of anesthesia influences outcome in mouse models of arterial thrombosis. Res. Pr. Thromb. Haemost..

[40] Walter D.H., Dimmeler S., Zeiher A.M. (2004). Effects of statins on endothelium and endothelial progenitor cell recruitment. Semin. Vasc. Med..

[41] Liu Y., Wei J., Hu L., Hu S. (2012). Beneficial effects of statins on endothelial progenitor cells. Am. J. Med. Sci..

[42] Luo J., Wei D., Li D., Wang L. (2018). Nitric oxide functions in stromal cell-derived factor-1-induced cytoskeleton changes and the migration of Jurkat cells. Oncol. Lett..

[43] Xu Y., Hu Y., Liu C., Yao H., Liu B., Mi S. (2018). A novel strategy for creating tissue-engineered biomimetic blood vessels using 3d bioprinting technology. Materials.

[44] Papaioannou T.G., Manolesou D., Dimakakos E., Tsoucalas G., Vavuranakis M., Tousoulis D. (2019). 3D bioprinting methods and techniques: Applications on artificial blood vessel fabrication. Acta Cardiol. Sin..

